# LncRNA coordinates Hippo and mTORC1 pathway activation in cancer

**DOI:** 10.1038/s41419-021-04112-w

**Published:** 2021-08-30

**Authors:** Shugeng Zhang, Shuhang Liang, Dehai Wu, Hongrui Guo, Kun Ma, Lianxin Liu

**Affiliations:** Department of General Surgery, Key Laboratory of Hepatosplenic Surgery, Ministry of Education, The First Affiliated Hospital of Harbin Medical University, Harbin, China

**Keywords:** Oncogenes, Long non-coding RNAs

## Abstract

The Hippo and mammalian target of rapamycin complex 1 (mTORC1) pathways are the two predominant pathways that regulate tumour growth and metastasis. Therefore, we explored the potential crosstalk between these two functionally relevant pathways to coordinate their tumour growth-control functions. We found that a Hippo pathway-related long noncoding RNA, *HPR*, directly interacts with Raptor, an essential component of mTORC1, to upregulate mTORC1 activation by impairing the phosphorylation of Raptor by AMPK. Knockdown or knockout of *HPR* in breast cancer and cholangiocarcinoma cells led to a reduction in tumour growth. Compared with *HPR* WT cells, *HPR-*overexpressing cells exhibited nuclear accumulation of YAP1, and significantly blocked the downregulation of mTORC1 signalling induced by energy stress. Thus, our study reveals a direct link between the Hippo and mTORC1 pathways in the control of tumour growth.

## Introduction

Cancer is the consequence of dysregulated signalling pathways. Dissecting the molecular mechanisms of cancer signalling pathways lays the foundation for developing targeted cancer therapies. YAP1 and TAZ are transcription factors/cofactors that regulate signature gene expression to control development and homoeostasis [1–4]. YAP1 has been demonstrated to facilitate the proliferation, invasion and epithelial–mesenchymal transition of various types of cancer cells. As a consequence, hyperactivation of YAP1 and expression of YAP1 target genes promote cancer initiation, progression and drug resistance [5–7]. The YAP1 pathway is regulated by upstream Hippo tumour suppressors, including MST1/2 and LATS1/2. Recent research has shown that a variety of molecular mechanisms regulate the activity of YAP/TAZ [8–11]. Upon nutrient stress, low energy triggers AMPK activation, which leads to YAP1 phosphorylation and degradation [12–14]. In cancer cells, the negative regulatory role of AMPK could be uncoupled by unknown mechanisms, leading to hyperactivation of the YAP1 pathway.

The mammalian target of rapamycin (mTOR) signalling pathway has important role in regulating cell growth, metabolism and survival. mTOR complex 1 (mTORC1) and mTOR complex 2 (mTORC2) are two of the major complexes [15]. mTORC1 contains the catalytic mTOR subunit and regulatory subunits, including Raptor [16]. Reports have proposed that Raptor modulates the enzymatic activity of mTOR and the recruitment of substrates, such as S6K and 4E-BP1 [17, 18]. mTORC1 plays essential roles in promoting cell growth and proliferation by regulating protein biosynthesis, autophagy, lipid biogenesis, mitochondrial metabolism and other pathways. The activity of mTORC1 is regulated by the integration of intracellular and extracellular stimuli, such as growth factors, nutrition and energy stress [19].

Recent advances in genome-wide transcriptome profiling have indicated the vast transcription of lncRNAs [20–22]. The upregulation of lncRNAs in cancer suggests the potential functional role of lncRNAs in modulating cellular activities, such as antiapoptosis, cancer metabolism reprogramming, antigen presentation, transcription regulation, mitochondrial metabolism and drug resistance [23–25]. Recently, lncRNAs have been demonstrated to play important roles in modulating cancer signalling pathways through RNA–protein interactions [11, 26–30]. Hence, lncRNAs may act as key signalling mediators that regulate cancer signalling cascades, which leads to malignancies. Here, we report that in breast cancer and cholangiocarcinoma, the YAP1 pathway and mTORC1 pathway are coactivated. Mechanistically, the expression of the lncRNA *HPR* is upregulated in breast cancer and cholangiocarcinoma compared with normal tissues. *HPR* is directly involved in YAP1 activation and associated with both the HEAT and WD40 domains of Raptor, and it regulates the enzymatic activity of mTORC1. Knocking down or genetically depleting *HPR* increases the phosphorylation of Raptor at Ser792, which is repressed in the presence of exogenous *HPR*. Knockdown or knockout of *HPR* in breast cancer and cholangiocarcinoma cells leads to a reduction in tumour growth. Compared with *HPR* WT cells, *HPR*-overexpressing cells exhibited an accumulation of YAP1 in the nucleus and significantly blocked the downregulation of mTORC1 signalling induced by energy stress. Hence, our research work demonstrates a lncRNA directed molecular mechanism for coactivation of the Hippo and mTORC1 pathways in cancer.

## Result

### Coactivation of the Hippo and mTORC1 pathways in cancer

**Fig. 1:**
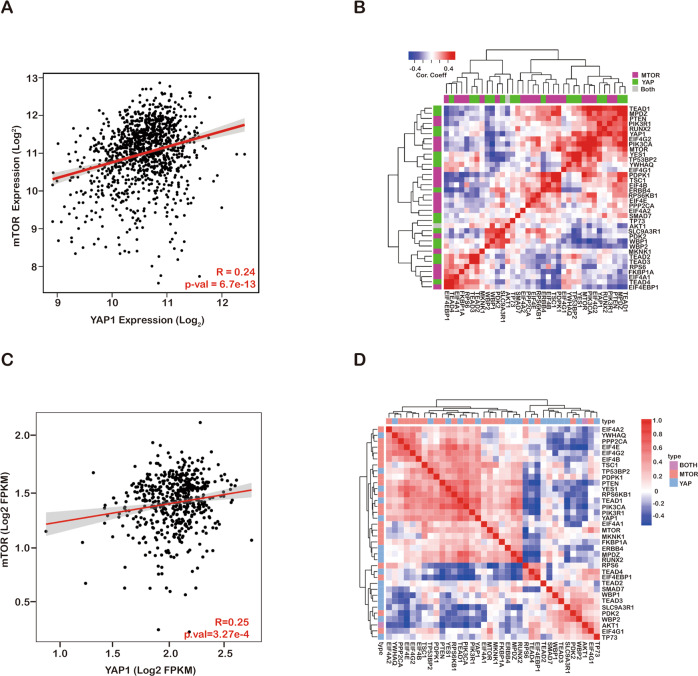
Coactivation of the Hippo and mTOR pathways in cancer. **a** Pearson’s correlation analysis comparing the expression of YAP1 and mTOR in TCGA database breast cancer tissues and Fisher’s exact test. **b** Pearson’s correlation analysis comparing mTOR pathway factors and the expression of YAP target genes in TCGA database breast cancer tissues, Fisher’s exact test. **c** Pearson’s correlation analysis comparing the expression of YAP1 and mTOR in TCGA database colon cancer tissues and Fisher’s exact test. **d** Pearson’s correlation analysis comparing the mTOR pathway factors and YAP target gene expression in TCGA database colon cancer tissues and Fisher’s exact test.

**Fig. 2:**
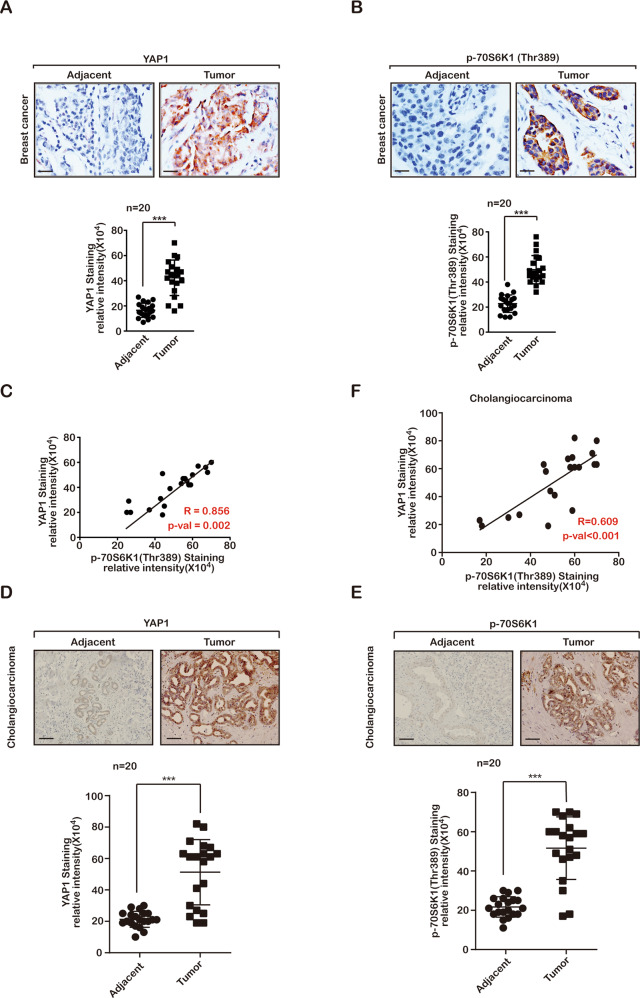
Coactivation of the Hippo and mTORC1 pathways in cancer. **a**, **b** Immunohistochemical staining using antibodies against YAP1 (**a**) and phospho-p70S6K1 (**b**) in human breast cancer tissues. Upper panel: representative images (scale bars, 100 µm); lower panel: statistical analysis of immunohistochemical staining (***P < 0.001). The results are the mean ± s.e.m. of *n* = 3 independent experiments. *P* values were determined by one-way ANOVA. **c** Pearson’s correlation analysis comparing staining density between YAP1 and phospho-p70S6K1, *n* = 20. **d**, **e** Immunohistochemical staining using antibodies against YAP1 (**d**) and phospho-p70S6K1 (**e**) in human cholangiocarcinoma tissues. Upper panel: representative images (scale bars, 400 µm); lower panel: statistical analysis of immunohistochemical staining (***P < 0.001). The results are the mean ± s.e.m. of *n* = 3 independent experiments. *P* values were determined by one-way ANOVA. **f** Pearson’s correlation analysis comparing staining density between YAP1 and phospho-p70S6K1, *n* = 20.

Both the Hippo and mTORC1 pathways are significantly upregulated in cancer [31, 32], hence, we performed a correlation analysis of the mRNA expression of Hippo and mTORC1 pathway-related genes in the TCGA database. The findings showed that the expression of Hippo and mTORC1 pathway genes is significantly correlated in breast and colon cancer (Fig. 1a–d). Using a tumour tissue microarray, we validated the status of the key factors in the Hippo and mTORC1 pathways using phosphorylated YAP1 and p70S6K. Our data indicated that YAP1 and phosphorylated 70S6K (Thr389) exhibited high positive rates compared with paired adjacent normal tissues in breast cancer, cholangiocarcinoma and colon cancer (Fig. 2a, b, d, e and Supplement Figs. 1A, 1B). Furthermore, the status of nuclear YAP1 overlapped with the positive staining of phospho-70S6K (Fig. 2c, f and Supplement Fig. 1C). Therefore, our data suggested that the Hippo and mTORC1 pathways are coactivated in cancer.

### LncRNA *HPR* is involved in YAP1 activation

**Fig. 3:**
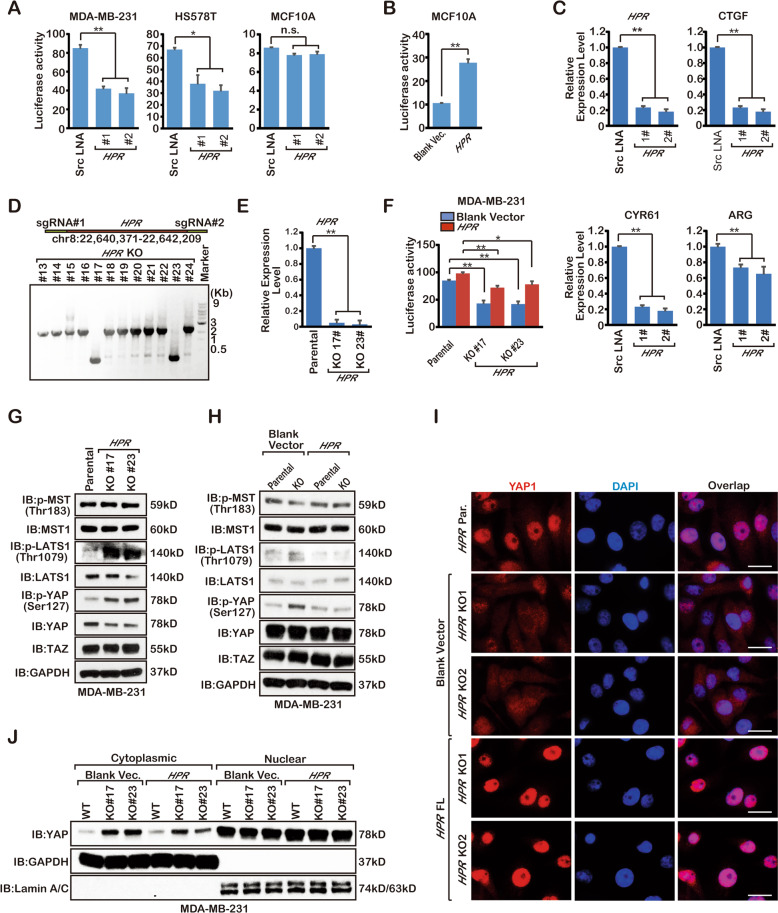
*HPR* regulates Hippo pathway activation. **a** Reporter assay detection of the activation of YAP1 in MDA-MB-231, HS578T and MCF10A cells transfected with the LNA of *HPR* (*P < 0.05, **P < 0.01). The results are the mean ± s.e.m. of *n* = 3 independent experiments. *P* values were determined by one-way ANOVA. **b** Reporter assay detection of the activation of YAP1 in MCF10A cells transfected with *HPR* cDNA (*P < 0.05, **P < 0.01). The results are the mean ± s.e.m. of *n* = 3 independent experiments. *P* values were determined by one-way ANOVA. **c** qPCR detection of *HPR* and substrates of YAP1 expression (**P < 0.01). The results are the mean ± s.e.m. of *n* = 3 independent experiments. *P* values were determined by one-way ANOVA. **d** CRISPR-Cas9 knockout of *HPR* in MDA-MB-231 cells. **e** qPCR detection of *HPR* expression in the MDA-MB-231 cells (**P < 0.01). The results are the mean ± s.e.m. of *n* = 3 independent experiments. *P* values were determined by one-way ANOVA. **f** Reporter assay detection of the activation of YAP1(*P < 0.05, **P < 0.01). The results are the mean ± s.e.m. of *n* = 3 independent experiments. *P* values were determined by one-way ANOVA. **g**, **h** Immunoblotting detection using the indicated antibodies in *HPR*-knockout MDA-MB-231 cells. Three independent experiments were performed and yielded similar results. **i** Immunofluorescence detection using indicated antibodies in the *HPR-*knockout MDA-MB-231 cells. **j** Immunoblotting detection using the indicated antibodies in *HPR-*knockout MDA-MB-231 cells. Three independent experiments were performed and yielded similar results.

To identify cancer-relevant lncRNAs that might be involved in the YAP1 signalling pathway, a previous study transfected the human Lincode siRNA library into MCF-7 cells that were engineered with a TEA domain transcription factor TEAD-driven luciferase reporter and subsequently determined the relative YAP1 activity [11]. More than 40 lncRNAs were potentially required for YAP1-dependent transcription. However, how these candidates regulate the YAP1 activation is still unclear. To further investigate whether these candidates were involved in YAP1 activation, we targeted lncRNAs to MCF10A, MDA-MB-231 and HS578T cells using locked nucleic acids (LNAs) together with the TEAD reporter 8xGTIIC-luciferase. Consistent with other studies, YAP1-dependent transcription activation was high in MDA-MB-231 and HS578T breast cancer cells compared with the MCF10A normal cells (Supplemental Fig. 2). Interestingly, knockdown of lncRNA-*FLG14107* dramatically downregulated YAP1 activation in breast cancer cells but not normal cells, thus, we renamed this lncRNA hippo pathway-related lncRNA (*HPR*) (Fig. 3a). However, YAP activation upregulated in the *HPR*-overexpressing MCF10A cells (Fig. 3b). Furthermore, the expression level of YAP1 substrates was also downregulated in the *HPR-*knockdown breast cancer cell lines (Fig. 3b). To further confirm that *HPR* was associated with the YAP1 signalling pathway, we used CRISPR-Cas9 technology to generate two *HPR-*knockout MDA-MB-231 cell clones (Fig. 3d), qRT-PCR data showed that *HPR* was not expressed in these two clones (Fig. 3e). We next tested whether *HPR* is required for YAP1 activation in this specific signalling context, and found that *HPR* depletion significantly impaired TEAD luciferase activity (Fig. 3f). The phosphorylation of LATS1 (Thr1079) and YAP1 (Ser127) was all increased following *HPR* knockout (Fig. 3g). Furthermore, we expressed full-length *HPR* in *HPR* KO cells for rescue experiments. We found that the expression of *HPR* decreased the phosphorylation of LATS1 (Thr1079) and YAP1 (Ser127) (Fig. 3h). At the same time, the expression of *HPR* restored YAP1 nuclear localisation (Fig. 3i, j). Thus, we found that *HPR* was associated with the Hippo pathway and regulated YAP1 activation.

### LncRNA *HPR* is associated with cancer

**Fig. 4:**
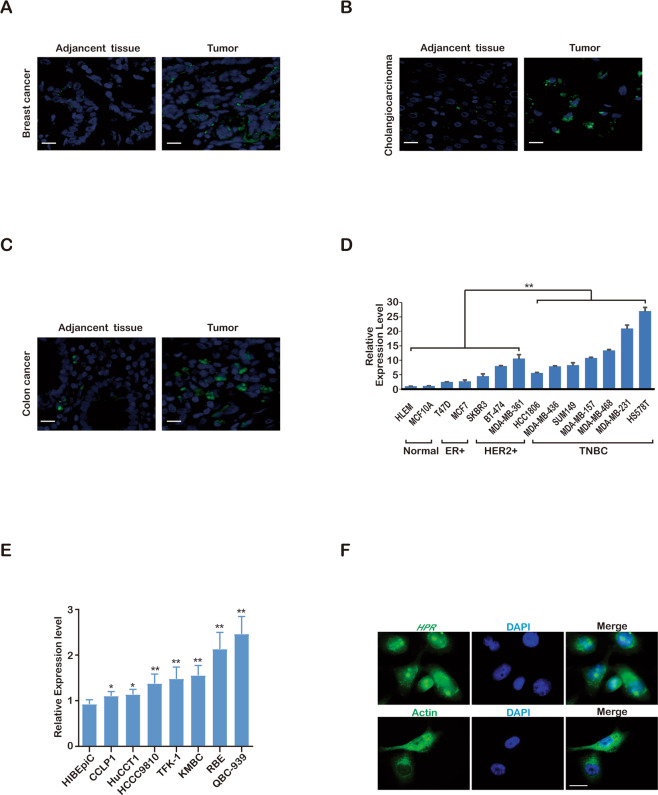
*HPR* is correlated with cancer. **a** RNA FISH detection of *HPR* expression in human breast cancer and adjacent normal tissues (scale bars, 50 µm). **b** RNA FISH detection of HPR expression in human cholangiocarcinoma and adjacent normal tissues (scale bars, 50 µm). **c** RNA FISH detection of HPR expression in human colon cancer and adjacent normal tissues (scale bars, 50 µm). **d** qPCR detection of *HPR* expression in a panel of breast cancer cell lines (**P < 0.01). The results are the mean ± s.e.m. of *n* = 3 independent experiments. *P* values were determined by two-way ANOVA. **e** qPCR detection of *HPR* expression in a panel of cholangiocarcinoma cell lines (*P < 0.05, **P < 0.01). The results are the mean ± s.e.m. of *n* = 3 independent experiments. *P* values were determined by one-way ANOVA. **g** RNA FISH detection of the *HPR* location in MDA-MB-231 cells.

We assumed that *HPR* may be expressed at lower levels in normal cells than in cancer cells, thus, to identify the mechanisms underlying the above discrepancies, we performed RNA in situ hybridisation on breast cancer tissue microarrays using RNA fluorescence in situ hybridization (FISH) technology to test the *HPR* expression level and examine the potential correlation of *HPR* with cancer. Our data indicated that YAP1 and phosphorylated 70S6K (Thr389) exhibit high positive correlations compared with paired adjacent normal tissues in breast cancer, cholangiocarcinoma and colon cancer (Fig. 4a–c). We also examined the expression of *HPR* in a panel of breast cancer cell lines and identified a higher expression of *HPR* in breast cancer cell lines than in normal cell lines (Fig. 4d). To detect whether *HPR* was highly expressed in other cancer types, we also examined the expression of *HPR* in cholangiocarcinoma cell lines and found higher expression of *HPR* in cholangiocarcinoma cell lines than in normal bile duct cells (Fig. 4e). We also employed the RNA FISH assay to analyse *HPR* expression in normal and cancer tissues from multiple organs and observed increased *HPR* expression in many types of human cancer tissues, including colorectal, lung and oesophageal tissues, compared with normal tissues (Table 1). Taken together, these results demonstrated the strong correlation of *HPR* expression with cancer progression and the relevance of elevated *HPR* expression to human cancer development and progression. We examined the subcellular localisation of *HPR* by RNA FISH and found that the *HPR* transcript was localised in the cytoplasm and nucleus (Fig. 4F).

**Table 1:** Reporter assay detection of YAP1 activation in the different cell lines.

Organ	Malignancy	Normal tissues
Vulva	+/−	Normal vulva −
Ovary	Squamous cell carcinoma +	Normal ovary −
Stomach	Adenocarcinoma −	normal stomach −
Oesophagus	Adenocarcinoma +	Normal oesophagus −
skin	Squamous cell carcinoma +	Normal skin −
Testis	Malignant melanoma ++	Normal testis +/−
Thyroid gland	Seminoma −	Normal thyroid gland −
Lung	Adenocarcinoma ++	Normal lung −
Liver	Squamous cell carcinoma −	Normal liver (cirrhosis) −
Kidney	Hepatocellular carcinoma ++	Normal kidney +/−
Skeletal muscle	Clear cell carcinoma −	Normal skeletal muscle −
Colon	Fibrosarcoma of buttocks ++	Normal colon +/−
Breast	Adenocarcinoma ++	Normal breast −
Rectum	Invasive ductal carcinoma +++	Normal rectum −
	Adenocarcinoma	

### *HPR* interacts with Raptor

**Fig. 5:**
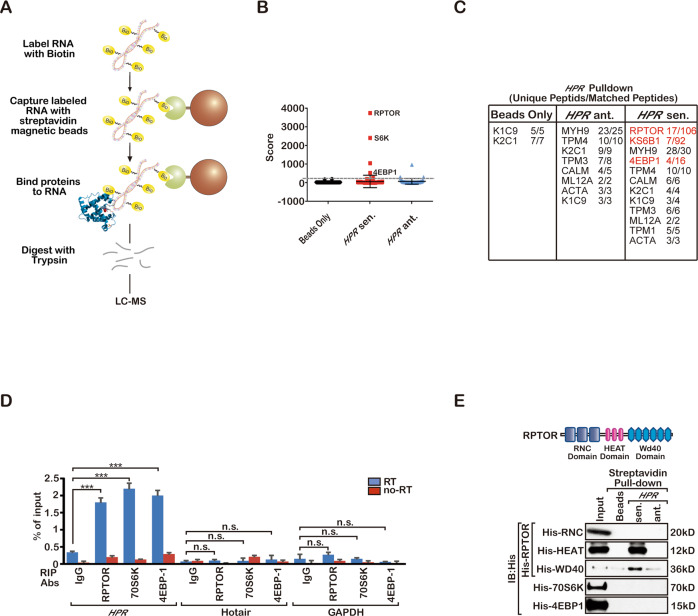
*HPR* interacts with Raptor. **a** Graphic illustration of the RNA pulldown assay. **b**, **c** List of the top *HPR*-associated proteins identified by RNA pulldown and MS analysis in MDA-MB-231 cells. **d** RIP assay detection of the interaction of *HPR* with the indicated proteins (***P < 0.001). The results are the mean ± s.e.m. of *n* = 3 independent experiments. *P* values were determined by one-way ANOVA. **e** In vitro RNA-protein binding assay detection of *HPR* interactions with the indicated proteins. Three independent experiments were performed and yielded similar results.

To identify how *HPR* regulates YAP1 activation, we performed RNA pulldown experiments using MDA-MB-231 cell lysates to identify whether *HPR* binds Hippo-YAP1 pathway proteins (Fig. 5a). Interestingly, *HPR* binds Raptor, S6K and 4E-BP1 but does not bind any Hippo-YAP1 pathway proteins, suggesting that it may mediate mTORC1 pathway activation. *HPR* binding to Raptor was confirmed by an in vivo RIP assay and an in vitro pulldown assay (Fig. 5b–d). Further study revealed that we find out *HPR* associates with both the HEAT and WD40 domains of Raptor, and regulates the enzymatic activity of mTORC1 (Fig. 5e).

### *HPR* regulates YAP1 and mTORC1 activation under cellular energy stress conditions

**Fig. 6:**
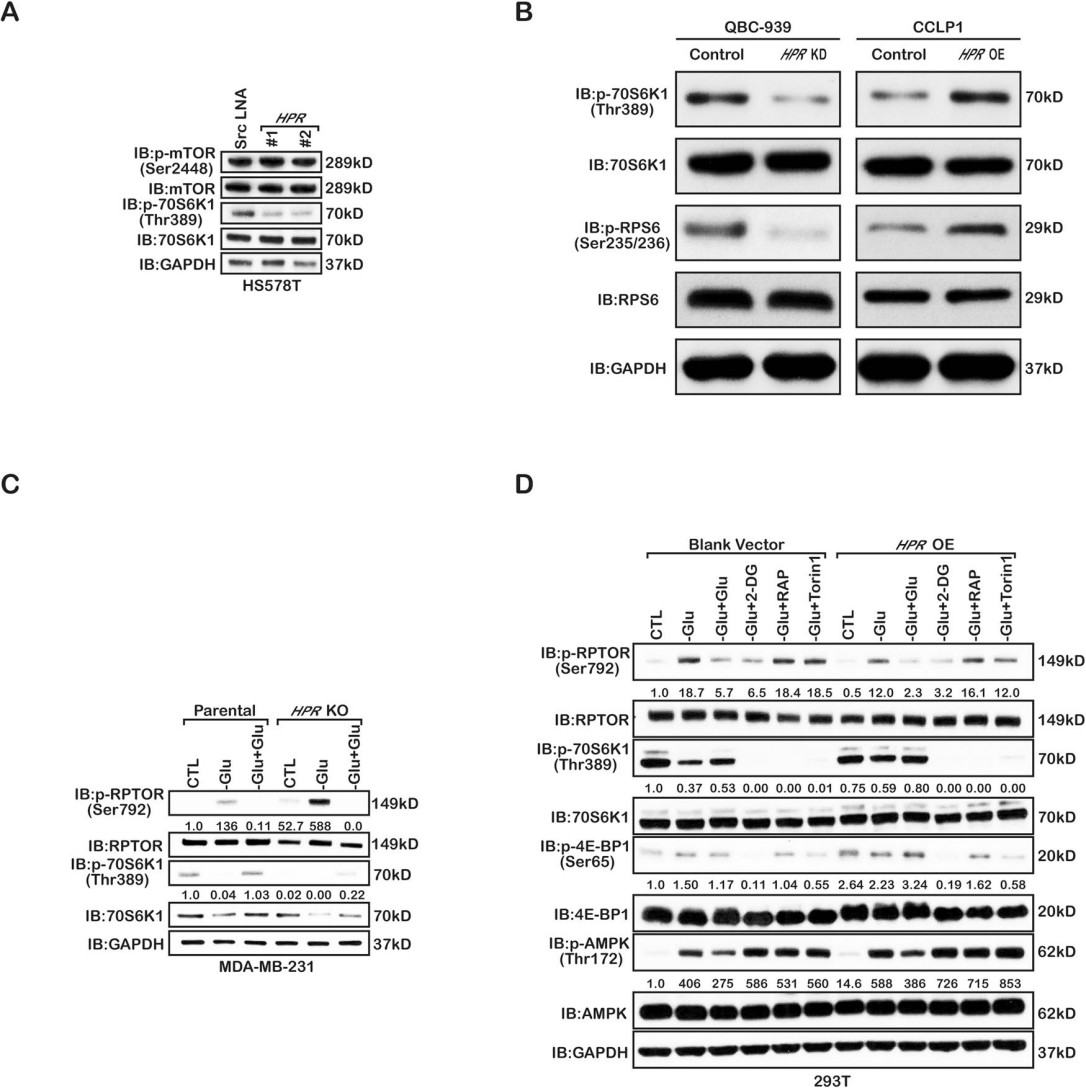
*HPR* regulates the activation of mTORC1. **a** Immunoblotting detection using the indicated antibodies in *HPR*-knockdown MDA-MB-231 cells. Three independent experiments were performed and yielded similar results. **b** Immunoblotting detection using the indicated antibodies in *HPR*-knockout MDA-MB-231 cells. Three independent experiments were performed and yielded similar results. **c** Immunoblotting detection using indicated antibodies in *HPR*-knockdown QBC-939 cells and in *HPR-*overexpressing CCLP1 cells. Three independent experiments were performed and yielded similar results. **d** Immunoblotting detection using the indicated antibodies in the 293 T cells. Three independent experiments were performed and yielded similar results.

Tumour cell growth is an energy-consuming process and must be coordinated with cellular energy status. Given the key function of the mTORC1 pathway in tumour cell growth regulation, we investigated whether mTORC1 is regulated by *HPR* under energy stress. We tested mTORC1 activation in the *HPR*-knockdown breast cancer cells and found that *HPR* depletion significantly decreased the phosphorylation of 70S6K (Thr389) but not mTOR (Ser2448) (Fig. 6a). Besides, the phosphorylation of 70S6K (Thr389) and the phosphorylation of RPS6 (Ser235/236) were also drastically decreased in cholangiocarcinoma (Fig. 6b). This result suggests that *HPR* does not regulate mTORC1 activation through direct regulation of the level of phosphorylated mTOR. To further verify the coordinated connection between the Hippo and mTORC1 pathways, we tested the activities of YAP1 when treating the *HPR*-overexpressing cell line with rapamycin (Supplement Fig. 4). The results revealed that *HPR* affects both Hippo and mTORC1 signalling activities in two independent ways. Recent studies have shown that mTORC1 activation can be downregulated under energy stress through direct AMPK directly phosphorylation of Raptor at site 792. Interestingly, we found that deletion of *HPR* significantly increased the level of phosphorylated Raptor (Ser792) under glucose starvation (Fig. 6c). Similarly, overexpression of *HPR* blocked AMPK phosphorylation of Raptor under glucose starvation (Fig. 6d). Interestingly, two recent studies found that AMPK phosphorylated YAP1 under glucose starvation and then inhibited YAP1 nuclear localisation. These data suggest that *HPR* may block AMPK phosphorylation of YAP1 and Raptor and then promote tumour cell growth under energy stress.

### *HPR* promotes cancer growth through the coactivation of YAP and mTORC1

**Fig. 7:**
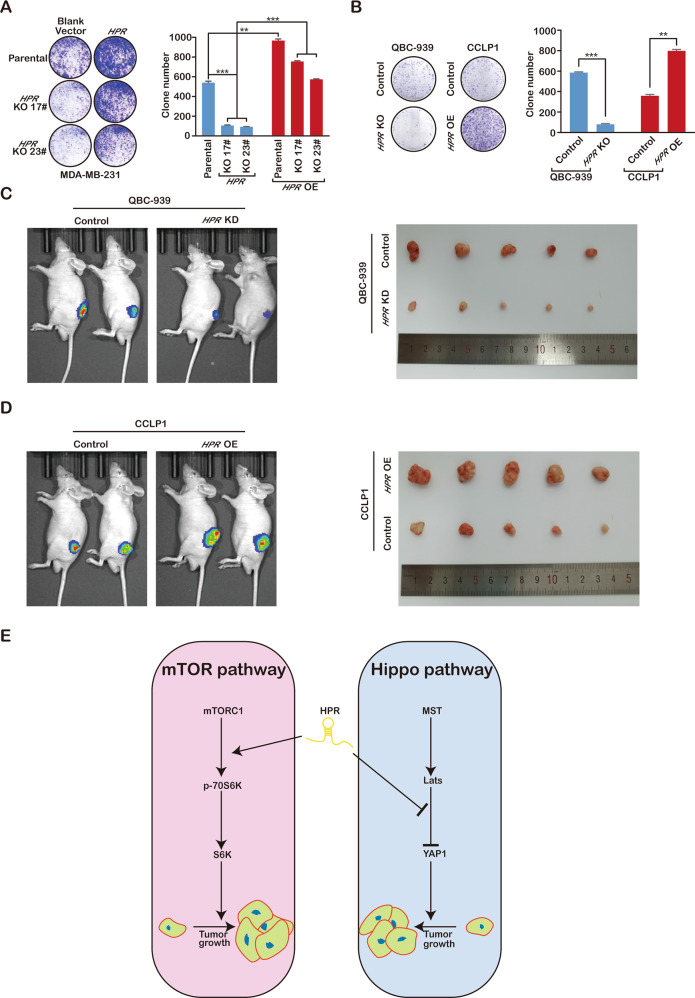
*HPR* inhibits the growth of cancer. **a** Clonogenicity assay detection of the growth of *HPR*-knockout MDA-MB-231 cells. **b** Clonogenicity assay detection of the growth of *HPR-*knockdown QBC-939 cells and overexpressed CCLP1 cells. **c** In vivo analyses of tumour growth in mice that were subcutaneously injected with *HPR*-knockdown QBC-939 cells. **d** In vivo analyses of tumour growth in mice that were subcutaneously injected with *HPR-*overexpressing CCLP1 cells. **e** Graphic illustration of the functional role of *HPR* coordinates the activation of the Hippo and mTORC1 pathways in cancer.

To determine whether *HPR* influences tumour growth, we examined the clonogenicity of an *HPR* KO cell line. SgRNA-mediated knockout of *HPR* expression significantly decreased MDA-MB-231 cell clonogenicity, and re-expression of full-length *HPR* rescued the cell growth ability (Fig. 7a). Similar to its effects in breast cancer, *HPR* overexpression notably increased the colony number in CCLP1 cells, whereas *HPR* silencing resulted in fewer colonies in QBC-939 cells (Fig. 7b). To test tumour growth in vivo, a subcutaneous xenograft mouse model was established to observe the role of *HPR* in cholangiocarcinoma, and the results supported that tumour volume was increased in the CCLP1-*HPR* group compared with that in the control group and that the tumour volume was decreased in mice inoculated with QBC-939-sh*HPR* cells (Fig. 7c, d). These results demonstrate that *HPR* may promote tumour growth through coactivation of YAP and mTORC1 and that targeting the *HPR* using LNA can suppress breast cancer in a preclinical model (Fig. 7d and Supplement Fig. 5).

## Discussion

The development of effective preventative and therapeutic strategies for cancer relies on a comprehensive understanding of the molecular mechanisms of the disease. Our findings demonstrate that lncRNAs coregulate both the YAP1 and mTORC1 pathways in cancer. Through large-scale screening of siRNAs targeting human long noncoding RNAs, we identified that one lncRNA, named *HPR*, can regulate YAP1 activation. Interestingly, *HPR* can also promote mTOR-dependent phosphorylation of S6K and 4E-BP1 by directly binding to Raptor, an important component of the mTORC1 complex. We also revealed that *HPR* blocks S792 phosphorylation of Raptor by AMPK under energy stress. The activation of the YAP and mTOR pathways is correlated in human cancer tissues. These findings suggest that *HPR* is important for co-regulating mTOR and YAP activation in cancer.

Cellular growth, proliferation and survival are critical processes that must be finely regulated to preserve the structure and function of different organs. The mechanistic targets of the rapamycin (mTOR) and Hippo pathways have recently emerged as the major signalling transduction cascades regulating organ size and cellular survival. The mTOR pathway promotes protein synthesis, cellular growth and survival. Although the Hippo pathway exerts opposite effects by inhibiting the cellular growth and proliferation and inducing cell death. These findings imply that tight coordination of these two pathways is important for the regulation of organ size and cellular integrity and strongly suggest the existence of multiple crosstalk mechanisms between the mTOR and Hippo signalling cascades. Although a recent study showed that YAP1 mediates crosstalk between the Hippo and PI3K-TOR pathways by suppressing PTEN via miR-29 [33], some PTEN-deficient cancer cells and tumour tissues also showed coactivation of the YAP and mTOR pathways. We also found that the knockdown of *HPR* increased T1029 phosphorylation of LATS. We first considered that mTORC1 may regulate LATS activation, because both pathways are regulated by *HRP*. In *HRP*-overexpressing CCLP1 cells, mTORC1 activity, as indicated by the decreased p70S6K1 T389 phosphorylation by the rapamycin treatment (Supplementary Figure 4), suggesting that *HPR* did not regulate mTORC1 activation through direct regulation of the level of phosphorylated mTOR. On the other hand, Hippo pathway activation was not affected by rapamycin-treated *HRP*-overexpressing CCLP1 cells (Supplementary Figure 4), indicating that mTORC1-independent pathways are involved in the regulation of LATS kinase activity in response to the manipulation of *HRP* expression. Therefore, the regulatory mechanisms that involve direct crosstalk between the Hippo and mTOR pathways remain unknown.

We believe that the molecular mechanisms presented in our study have broad biological implications. In conditions where nutrients are scarce, AMPK acts as a metabolic checkpoint that inhibits cellular growth. AMPK regulates cell growth by suppressing mTORC1 pathway via phosphorylated TSC2 and RAPTOR. Interestingly, two recent studies found that AMPK directly phosphorylated YAP1 and retained the cytoplasmic localisation of YAP1 under glucose starvation conditions [12, 13]. As nutrient concentrations in tumours are different from those in normal tissues, cancer cells in vivo may have metabolic dependencies that are not shared by normal cells. In particular, tumour glucose concentrations are frequently three- to tenfold lower than those in non-transformed tissues. These data suggest that some factors can coregulate mTORC1 and YAP1 activation and promote tumour growth under low glucose concentrations. Therefore, our study reveals that the lncRNA *HPR*, which shows high tumour expression, may play a role in tumour growth by co-regulating mTORC1 and YAP1 activation.

## Methods

### Clinical samples

The primary tumour and some paired normal tissues were obtained from individuals with breast, cholangiocarcinoma and colon cancer diagnosed at the First Affiliated Hospital of Harbin Medical University. The protocol was approved by the First Affiliated Hospital of Harbin Medical University Research Ethics Committee. All tissue samples were collected in compliance with the informed consent policy.

### Animal studies

All animal experiments were performed in accordance with a protocol approved by the Institutional Animal Care and Use Committee of the First Affiliated Hospital of Harbin Medical University. Luciferase-labelled QBC-939 and CCLP1 cells (5 × 10^6^) were suspended in 150 μL PBS to be subcutaneously injected into the flanks of mice. Tumour growth was examined by bioluminescent imaging every week using an IVIS Spectrum Xenogen Imaging System (Caliper Life Sciences) at the Small Animal Imaging Facility of the First Affiliated Hospital of Harbin Medical University.

### Cell culture, transfection and treatments

The human normal biliary cell line HIBEpiC was obtained from ScienCell Research Laboratories (Carlsbad, CA, USA). The HuCCT1 cell line was kindly provided by the Cancer Cell Repository of the Tohoku University in Japan. The CCLP1 and KMBC cell lines were purchased from BeNa Culture Collection (Beijing, China). The RBE, QBC-939 and HCCC9810 cell lines were purchased from Shanghai Bioleaf Biotech Corporation (Shanghai, China). The RBE, HCCC9810, QBC-939, CCLP1 and HuCCT1 were authenticated using short tandem repeat (STR) analysis. The human breast cancer cell lines MDA-MB-231, HS578T and the human embryonic kidney cell line HEK293T were purchased from American Type Culture Collection (ATCC) and maintained in Dulbecco’s modified essential medium (DMEM) supplemented with 10% fetal bovine serum (FBS) at 37°C in 5% CO_2_ (v/v). MCF10A cells were maintained in DMEM/F12 medium supplemented with 5% horse serum, 200 ng/ml epidermal growth factor, 500 ng/ml hydrocortisone, 100 ng/ml cholera toxin and 10 µg/ml insulin at 37°C in 5% CO2 (v/v). For the glucose treatment, transfected plasmids or *HRP* KO cells were seeded in proper dishes. Twenty-four hours later, the cells were washed and refreshed with glucose-free DMEM with 10% dialysed FBS. Four hours later, the cells were treated with d-glucose (25 mM), 2-DG (25 mM), rapamycin (10 nM) and Torin1 (10 nM) for 4 h in various experiments. For cell transfection, LNA transfections were performed using DharmaFECT4 (GE Healthcare). Plasmid transfections were performed using Lipofectamine 3000 (Invitrogen).

### Plasmid construction and recombinant proteins

The full-length Raptor mammalian expression vector was obtained from OriGene. Bacterial expression vectors for three Raptor domains (His_6_-RNC, His_6_-HEAT and His_6_-WD40) were constructed in the pET-DEST42 vectors using the Gateway system (Life Technologies). Full-length pGEM-3Z-*HPR* was purchased from GenScript Biotech. A mammalian expression vector for wild-type *HPR* was constructed by subcloning the gene sequences into pBabe (Addgene). The 8xGTIIC-luciferase vector was obtained from Addgene (Plasmid #34615). The following recombinant proteins were used in this study. CRISPR/Cas9 KO *HPR* plasmids were designed using four pairs of sgRNAs (oligonucleotide sequences and primers) to generate stable knockout cell lines of MDA-MB-231 cell lines (Gene Editing/Cellular Model Core Facility, MD Anderson Cancer Center). Full-length human S6K1 protein was purchased from Abcam (ab167933) and full-length human 4E-BP1 protein was purchased from Sigma (SRP5162-50UG). Recombinant His_6_-RNC, His_6_-HEAT and His_6_-WD40 proteins were expressed in *Escherichia coli* strain BL21-CodonPlus (DE3)-RIPL (Agilent Technologies) and purified using HisPur™ Cobalt Spin Columns (Life Technologies).

### Antibodies

Anti-phospho-MST1 (Thr183) rabbit mAb (3681), Anti-phospho-MST1 rabbit mAb (3682), anti-phospho-LATS1 (Thr1079) (D57D3) rabbit mAb (8654), anti-LATS1 (C66B5) rabbit mAb (3477), anti-phospho-YAP (Ser127) (D9W2I) rabbit mAb (13008), anti-YAP (D8H1X) rabbit mAb (14074), anti-TAZ (D3I6D) rabbit mAb (70148), anti-phospho-mTOR (Ser2448) (D9C2) rabbit mAb (5536), anti-mTOR (7C10) rabbit mAb (2983), anti-phospho-Raptor (Ser792) rabbit mAb (2083), anti-Raptor (24C12) rabbit mAb (2280), anti-phospho p70S6 Kinase (Thr389) (D5U1O) rabbit mAb (97596), anti-p70S6 Kinase (49D7) rabbit mAb (2708), anti-phospho 4E-BP1 (Ser65) (D9G1Q) rabbit mAb (13443), anti-4E-BP1 rabbit mAb (9452), anti-phospho AMPKα (Thr172) (D4D6D) rabbit mAb (50081), anti- AMPKα (D5A2) rabbit mAb (5831), anti-Lamin A/C (4C11) mouse mAb (4777) and anti-His-Tag (D3I1O) rabbit mAb (12698) were purchased from Cell Signaling Technology. Anti-GAPDH (6C5) mouse mAb (sc-32233) was purchased from Santa Cruz Biotechnology.

### Biotinylated RNA preparation

The *HPR* lncRNA sequence was cloned into the pGEM-3Z vector (Promega) for in vitro transcription using Biotin RNA Labelling Mix (Roche) and MEGAscript^®^ Transcription Kit (Life Technologies). Biotinylated RNAs were purified by RNA Clean & Concentrator™-5 (Zymo Research).

### RNA isolation, qRT-PCR, cell lysis, immunoprecipitation, immunoblotting and RNA immunoprecipitation (RIP) assay

Total RNA was isolated from cells using an RNeasy Mini Kit (QIAGEN) following the manufacturer’s protocol. First-strand cDNA synthesis from total RNA was carried out using iScript Reverse Transcription Supermix for RT-qPCR (Bio-Rad). Primer sequences are listed in the Oligonucleotide Sequences and Primers section. Cells were homogenised in 1× RIPA buffer (EMD Millipore) supplemented with Protease/Phosphatase Inhibitor Cocktail (Pierce, Thermo Scientific), Panobinostat (Selleck chemicals) and Methylstat (Sigma-Aldrich). Lysates were cleared by centrifugation at 13,000 rpm for 15 min at 4°C. Supernatants were analysed for immunoprecipitation with the indicated antibodies and the immunoprecipitated proteins were either subjected to immunoblotting or protein identification by mass spectrometry. RIP assay was performed as previously described [25].

### Immunohistochemistry and immunofluorescence

For immunohistochemistry, tumour tissues were fixed in neutral buffered formalin overnight and embedded in paraffin. Five-micrometre sections were baked for 30 min at 60 °C, and then deparaffinized. Antigen was retrieved at 98 °C for 20 min in 10 mM citrate buffer pH 6. Endogenous peroxidase activity was quenched by incubating the sections in 3% hydrogen peroxide solution for 10 min. All incubations were performed at room temperature unless otherwise stated. After blocking in 5% BSA/0.05% Tween-20, primary antibodies were applied (anti-rabbit YAP, 1:200; anti-rabbit phosphor-p70S6 kinase (Thr389), 1:200 and anti-rabbit phospho 4E-BP1 (Ser65)) and the sections were incubated overnight at 4°C. Subsequently, the sections were incubated with labelled polymer-HRP (Dako; K4065) for 30 min. For all staining, counterstaining with DAB positivity was analysed in five visual fields at ×200 magnification.

For immunofluorescence, cells were fixed with 4% PFA at 4 °C for 10 min at room temperature. The samples were blocked in 10% normal goat serum, and PBS with 0.25% Triton X-100 for 1 h at room temperature. Primary antibodies targeting YAP1 were incubated overnight at 4 °C in a blocking solution. After extensive washing in PBS-0.25% Triton X-100, the secondary antibody was added to the blocking solution and incubated for 1 h. After extensive washing in PBS-0.25% Triton X-100, nuclei were stained with DAPI (2 µg/ml; Life Technologies) for 10 min at room temperature. Slides were mounted with ProLong Gold anti-fade reagent (Life Technologies) and imaged with a confocal microscope (Zeiss).

### RNA fluorescence in situ hybridisation

RNA FISH was performed using LNA FISH technology according to the manufacturer’s instructions (Exiqon) with minor modifications, which have been described previously [34]. The probe is listed in the Oligonucleotide Sequences and Primers section.

### RNA pulldown and mass spectrometry analysis

RNA pulldown followed by a mass spectrometry analysis, and in vitro RNA-protein binding assay, and an in vitro RNA pulldown assays were performed as described previously [34]. In brief, the RNA-capture beads were incubated with recombinant proteins (1 µg): HIS-p70S6K and HIS-4E-BP1; and HIS-RNC, HIS-heat and HIS-WD40 in binding buffer [50 mM Tris-HCl pH 7.9, 10% glycerol, 100 mM KCl, 5 mM MgCl_2_, 10 mM β-ME 0.1% NP- 40] for 1 h at 30 °C.

### Reporter assay

To characterise TEAD activity in breast cancer cells and normal cells, the indicated transfected cells were transfected with the 8xGTIIC-Luc plasmids. Forty-eight hours later, luciferase luminescence was measured using the Dual-Glo luciferase assay kit (Promega)

### Oligonucleotide sequences and primers

#### Primers for RT-qPCR

*HPR* (5′-GAC CTG TGC ACT GCC AAA AG-3′ and 5′-CCA CCC CCT GCA TTC ATT CT-3′)*; CTGF* (5′-CTT GCG AAG CTG ACC TGG AAG A-3′ and 5′-CCG TCG GTA CAT ACT CCA CAG A-3′); *CYR61* (5′-GGA AAA GGC AGC TCA CTG AAG C-3′ and 5′-GGA GAT ACC AGT TCC ACA GGT C-3′); *ANKRD1* (5′-CAC TTC TAG CCC ACC CTG TGA-3′ and 5′-CCA CAG GTT CCG TAA TGA TTT-3′); *GAPDH* (5′-ATG GGG AAG GTG AAG GTC G-3′ and 5′-GGG GTC ATT GAT GGC AAC AAT A-3′).

#### RNA FISH probe

*HPR* (/56-FAM/5′-AAG TGA ATG AAC AGG CTG AGT-3′); *β*-actin (/Fluorescein/5′-CTC ATT GTA GAA GGT GTG GTG CCA-3′).

#### siRNA sequences

Control siRNA (5′-UAA GGC UAU GAA GAG AUA CUU-3′ and 5′-GUA UCU CUU CAU AGC CUU AUU-3′); *mTOR 1#* (5′-CUA GUG AAA UGC UGG UCA AUU-3′ and 5′-UUG ACC AGC AUU UCA CUA GUU-3′); *mTOR 2#* (5′-GCC UAU UCU GAA GGC AUU AUU-3′ and 5′-UAA UGC CUU CAG AAU AGG CUU-3′); *YAP1 1#* (5′-CAC CUA UCA CUC UCG AGA UUU-3′ and 5′-AUC UCG AGA GUG AUA GGU GUU-3′); *YAP1 2#* (5′-GCU CAU UCC UCU CCA GCU UUU-3′ and 5′-AAG CUG GAG AGG AAU GAG CUU-3′).

#### LNA GapmeR sequences

Negative control (5′-AAC ACG TCT ATA CGC-3′); *HPR* 1# (5′-GGA AGG TTA GGA TCG G-3′); *HPR* 2# (5′-GCA TCT GAT CGG AGT C-3′).

#### sgRNA sequences

*HPR* 1# (5′-GCC TGG GTT ATT GGA AGT CA-3′); *HRP* 2# (5′-GGA AAG ATG TGG GCC GTG CG-3′).

### Data analysis and statistics

Analyses of relative gene expression were determined using the 2-ΔΔ*C*t method with *GAPDH* as the internal reference gene. The results are reported as the mean ± standard error of the mean of at least three independent experiments. Each exact *n* values is indicated in the corresponding figure legend. Comparisons were performed using two-tailed paired Student’s *t* test or two-way analysis of variance (n.s. *p* > 0.05, *p < 0.05, **p < 0.01 and ***p < 0.001) as indicated in the individual figures. Pearson chi-square test or Fisher’s exact test was implemented for statistical analyses of the associations between markers and clinical parameters, as indicated in the individual figures.

## Supplementary information

Coactivation of the Hippo and mTORC1 pathways in colon cancer.

RNA FISH detection of HPR expression in different human cancer tissues.

qPCR detection of HPR expression in HPR-knockdown QBC-939 cells and HPR-overexpressing CCLP1 cells.

HPR regulates Hippo pathway activation independent of mTORC1 activation.

Coactivation of the Hippo and mTORC1 pathways in mouse tumour samples.

Reporter assay detection of YAP1 activation in the different cell lines.

supplement figure legends

author-contribution-form

the Reproducibility Checklist forms
