# Supplementary figures and images for: LncRNA coordinates Hippo and mTORC1 pathway activation in cancer
Source: Cell Death Dis. 2021 Aug 30;12(9):822. doi: 10.1038/s41419-021-04112-w (PMC8405608; doi:10.1038/s41419-021-04112-w)

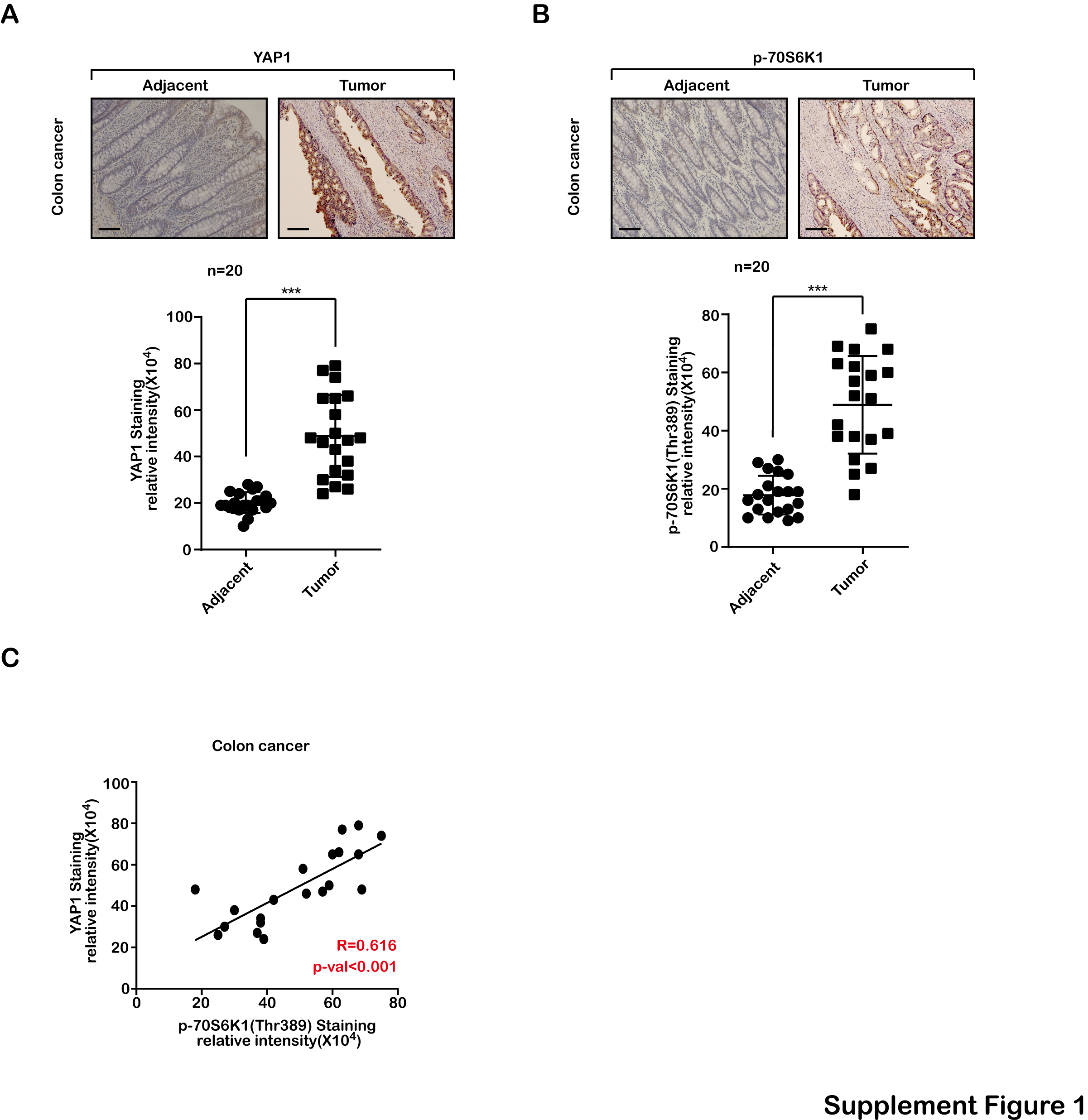

Supplement: Supplementary file 1 — Coactivation of the Hippo and mTORC1 pathways in colon cancer. [file 41419_2021_4112_MOESM1_ESM.tif]

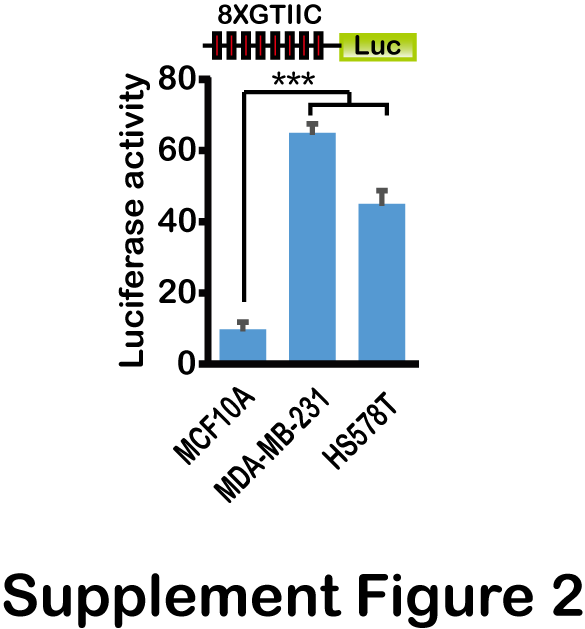

Supplement: Supplementary file 2 — RNA FISH detection of HPR expression in different human cancer tissues. [file 41419_2021_4112_MOESM2_ESM.tif]

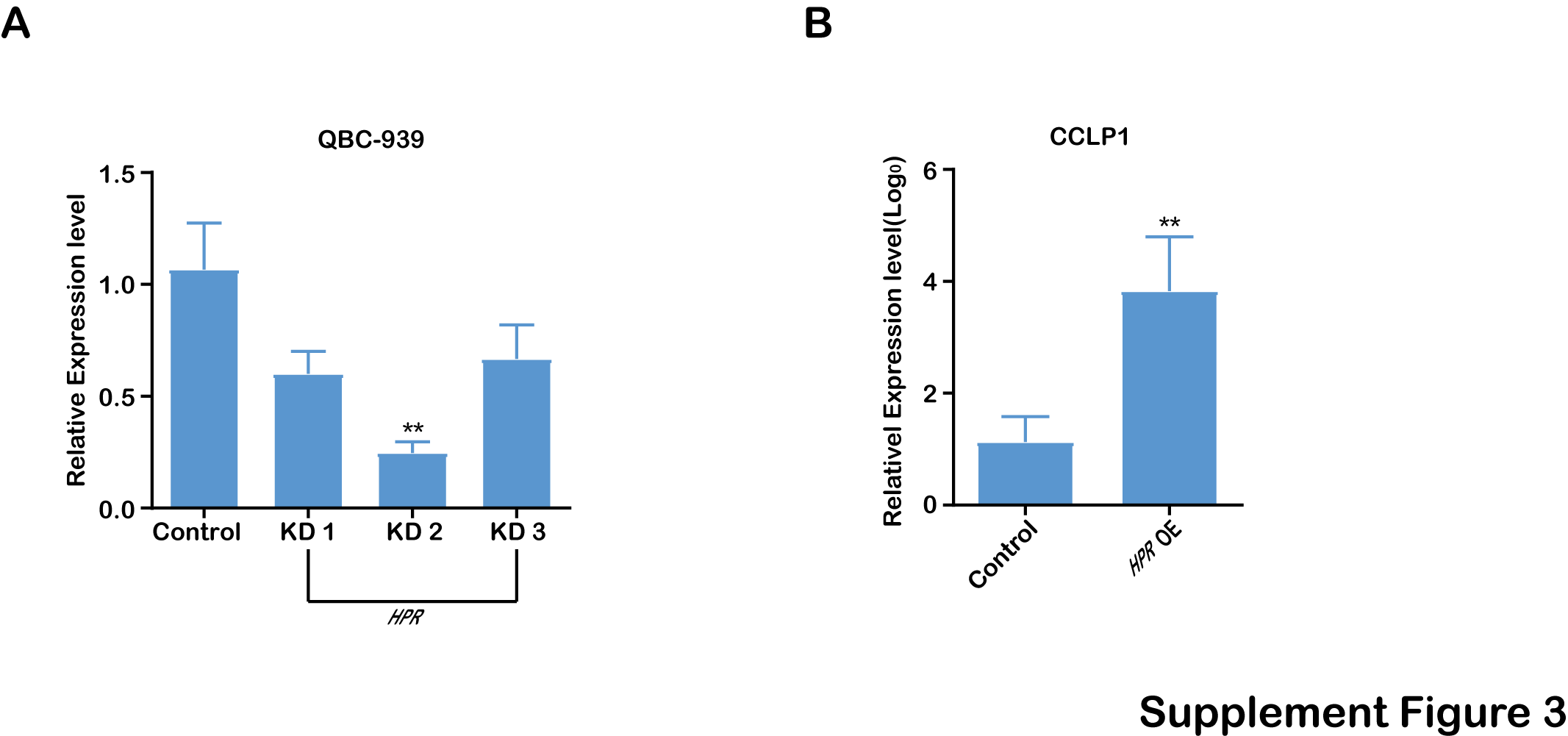

Supplement: Supplementary file 3 — qPCR detection of HPR expression in HPR-knockdown QBC-939 cells and HPR-overexpressing CCLP1 cells. [file 41419_2021_4112_MOESM3_ESM.tif]

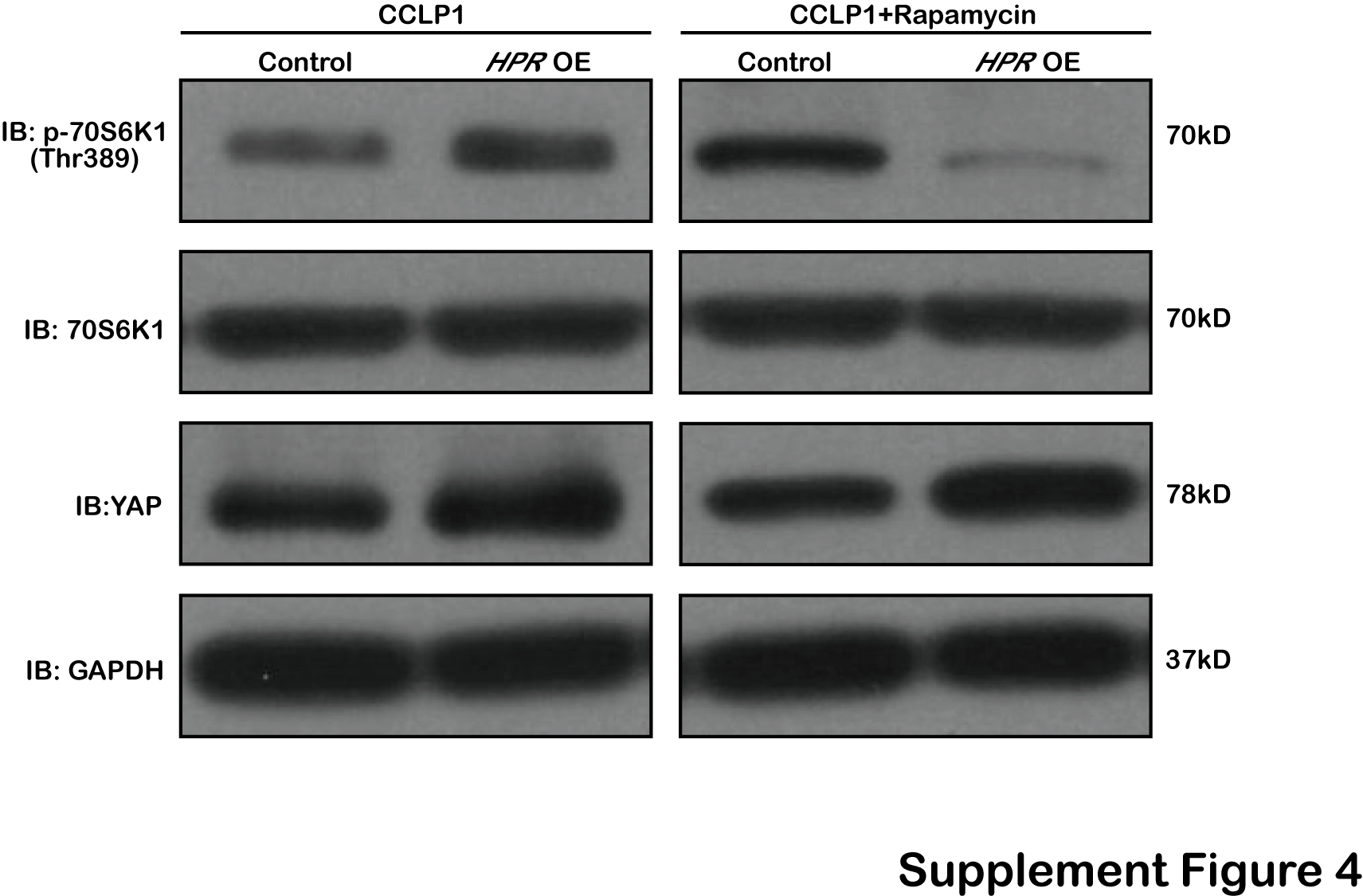

Supplement: Supplementary file 4 — HPR regulates Hippo pathway activation independent of mTORC1 activation. [file 41419_2021_4112_MOESM4_ESM.tif]

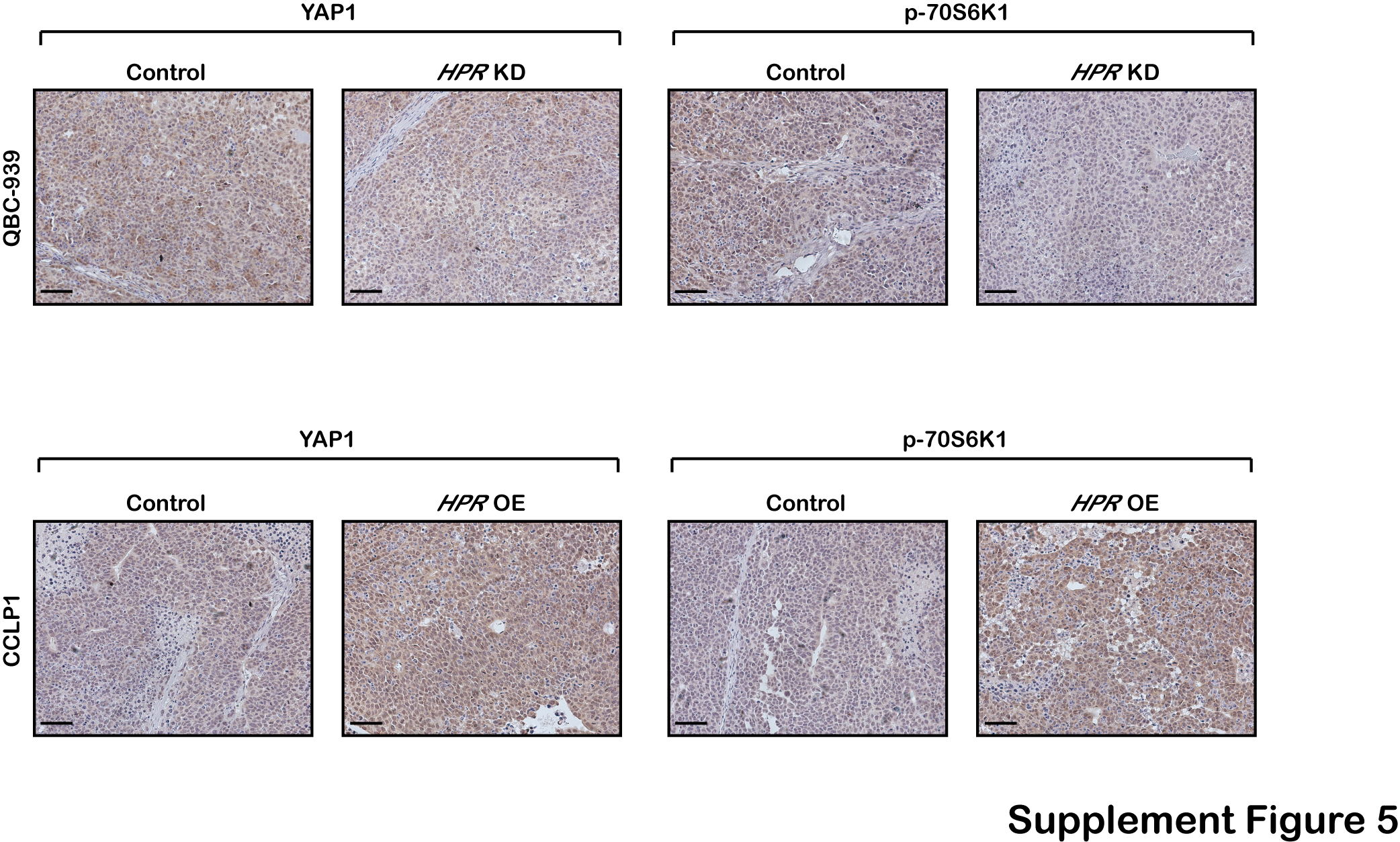

Supplement: Supplementary file 5 — Coactivation of the Hippo and mTORC1 pathways in mouse tumour samples. [file 41419_2021_4112_MOESM5_ESM.tif]
