# Supplementary material for: LncRNA coordinates Hippo and mTORC1 pathway activation in cancer
Source: Cell Death Dis. 2021 Aug 30;12(9):822. doi: 10.1038/s41419-021-04112-w (PMC8405608; doi:10.1038/s41419-021-04112-w)
Supplement: Supplementary file 6 — Reporter assay detection of YAP1 activation in the different cell lines. [file 41419_2021_4112_MOESM6_ESM.docx]

| Organ | Malignancy | Normal tissues |
| --- | --- | --- |
| Vulva  Ovary  Stomach  Esophagus  skin  Testis  Thyroid gland  Lung  Liver  Kidney  Skeletal muscle  Colon  Breast  Rectum | +/-  Squamous cell carcinoma +  Adenocarcinoma  **-**  Adenocarcinoma +  Squamous cell carcinoma +  Malignant melanoma ++  Seminoma -  Adenocarcinoma ++  Squamous cell carcinoma -  Hepatocellular carcinoma ++  Clear cell carcinoma -  Fibrosarcoma of buttocks ++  Adenocarcinoma ++  Invasive ductal carcinoma +++  Adenocarcinoma | Normal vulva -  Normal ovary -  normal stomach -  Normal esophagus -  Normal skin -  Normal testis +/-  Normal thyroid gland -  Normal lung -  Normal liver (cirrhosis) -  Normal kidney +/-  Normal skeletal muscle -  Normal colon +/-  Normal breast -  Normal rectum - |

**Table.1 Reporter assay detection of YAP1 activation in the different cell lines.**
