# Supplementary material for: LncRNA coordinates Hippo and mTORC1 pathway activation in cancer
Source: Cell Death Dis. 2021 Aug 30;12(9):822. doi: 10.1038/s41419-021-04112-w (PMC8405608; doi:10.1038/s41419-021-04112-w)
Supplement: Supplementary file 7 — supplement figure legends [file 41419_2021_4112_MOESM7_ESM.docx]

**Supplement Figure Legends**

**Supplement figure 1.** **Coactivation of the Hippo and mTORC1 pathways in colon cancer.** (a-b) Immunohistochemical staining using antibodies against YAP1 (a) and phospho-p70S6K1 (b) in human colon cancer tissues. Upper panel: representative images (scale bars, 400 µm); lower panel: statistical analysis of immunohistochemical staining (***P < 0.01). The results are the mean ± s.e.m. of n = 3 independent experiments. P values were determined by one-way ANOVA. (c) Pearson’s correlation analysis comparing the staining density between YAP1 and phospho-p70S6K1, n = 20.

**Supplement figure 2.** RNA FISH detection of *HPR* expression in different human cancer tissues. (***P < 0.01). The results are the mean ± s.e.m. of n = 3 independent experiments. P values were determined by one-way ANOVA.

**Supplement figure 3.** qPCR detection of *HPR* expression in *HPR* knockdown QBC-939 cells (a) and *HPR* overexpressing CCLP1 cells (b) (**P < 0.01). The results are the mean ± s.e.m. of n = 3 independent experiments. P values were determined by one-way ANOVA.

**Supplement figure 4.** ***HPR* regulates Hippo pathway activation independent of mTORC1 activation.** Immunoblotting detection using the indicated antibodies in *HPR* overexpressing CCLP1 cells with or without rapamycin treatment. Three independent experiments were performed and yielded similar results.

**Supplement figure 5.** **Coactivation of the Hippo and mTORC1 pathways in mouse tumour samples.** Immunohistochemical staining using antibodies against YAP1 and phospho-p70S6K1 in mouse subcutaneous tumours.
