# Supplementary material for: LncRNA coordinates Hippo and mTORC1 pathway activation in cancer
Source: Cell Death Dis. 2021 Aug 30;12(9):822. doi: 10.1038/s41419-021-04112-w (PMC8405608; doi:10.1038/s41419-021-04112-w)
Supplement: Supplementary file 8 — author-contribution-form [file 41419_2021_4112_MOESM8_ESM.pdf]

DECLARATION OF CONTRIBUTIONS TO ARTICLE

ADMC

Manuscript Number: CDDIS-21-0880

Journal Name: *Cell Death & Disease* (the 'Journal')

Proposed Title of the Contribution:

LncRNA Coordinates Hippo and mTORC1 Pathway Activation in Cancer (the 'Contribution)

Author(s):

Shugeng Zhang, Shuhang Liang, Dehai Wu, Hongrui Guo, Kun Ma, Lianxin Liu * (the 'Authors')

For all *CDDis* articles, each person named as an author in the published version must be able to show he or she has contributed substantially to the article.

Authorship credit should be based on 1) substantial contributions to conception and design, acquisition of data, or analysis and interpretation of data; 2) drafting the article or revising it critically for important intellectual content; and 3) final approval of the version to be published. Authors should meet conditions 1, 2 and 3.

Any person who cannot be shown to have made a substantial contribution to the article cannot be listed as an author in the final version. The name of any person who is deemed to have made a minor contribution can, however, appear in the Acknowledgments section of the article.

Please complete the table below to indicate the contributions of all named authors to the manuscript.

Author Full Name:	Specification of Contribution to the Manuscript:
Shugeng Zhang	analysis and interpretation of data,drafting the article,final approval of the version to be published
Shuhang Liang	analysis and interpretation of data; revising the article,final approval ofthe version to be published.
Dehai Wu	acquisition of data, revising the article, final approval ofthe version to be published.
Hongrui Guo	analysis of data; drafting the article,final approval ofthe version to be published.
Kun Ma	acquisition of data,drafting the article,final approval ofthe version to be published.
Lianxin Liu	conception and design, acquisition of data,drafting and revising the article,final approval of the version to be published.

Please complete the table below to indicate the contributions of all named authors to the figures.

Figure 1:

Shuhang Liang, Hongrui Guo and Kun Ma performed the bioinformatics analysis and drew the figure1a-1f.

Figure 2:

Lianxin Liu designed the experiments. Shugeng Zhang executed the primary studies and drew the figure2a-2e.

Figure 3:

Lianxin Liu conceived of the project and designed the experiments. Shugeng Zhang and Shuhang Liang performed related experiments,analyzed the data and made the figure 3a-3i.

Figure 4:

Lianxin Liu conceived of the project and designed the experiments. Shugeng Zhang and Dehai Wu performed related experiments,analyzed the data and drew the figure 4a-4f.

Figure 5:

Lianxin Liu conceived of the project and designed the experiments. Shugeng Zhang and Shuhang Liang performed related experiments,analyzed the data and drew the figure 5a-5e.

Figure 6:

Lianxin Liu conceived of the project and designed the experiments. Shugeng Zhang, Shuhang Liang and Dehai Wu performed related experiments,analyzed the data and drew the figure 6a-6d.

Signed for and on behalf of the Author(s):	Print Name:	Date:
Lianxin Liu	Lianxin Liu	2021/05/14
